# Methodology for Assessing University Members’ Health and Well-Being and Their Influencing Factors: Protocol for a Rapid Scoping Review of Survey-Based Research

**DOI:** 10.2196/83845

**Published:** 2026-03-31

**Authors:** Varga Zsuzsanna, László Balkányi, Anikó Gyulai, László Lippai, Andrea Mária Rucska†, Zsuzsanna Soósné Kiss, Péter Varsányi, József Vitrai, Boróka Gács

**Affiliations:** 1 Department of Behavioural Sciences Medical School University of Pécs Pécs, Baranya Hungary; 2 Syreon Research Institute Budapest Hungary; 3 Department of Preventive Health Sciences Faculty of Health Sciences University of Miskolc Miskolc, Borsod-Abaúj-Zemplén Hungary; 4 Institute of Applied Health Sciences and Environmental Education University of Szeged Szeged Hungary; 5 Faculty of Health Sciences University of Miskolc Miskolc Hungary; 6 Department of Obstetrics and Gynecology Faculty of Health and Sport Sciences Széchenyi István University Győr, Győr-Moson-Sopron Hungary; 7 Society to Renew the Hungarian Public Health Budapest Hungary; 8 Department of Psychology and Health Management Faculty of Sports and Health Sciences Széchenyi István University Győr, Győr-Moson-Sopron Hungary

**Keywords:** scoping review, health, well-being, university members, survey

## Abstract

**Background:**

This scoping review protocol addresses the imperative need for a comprehensive understanding of the health and well-being of university members, aligning with the global recognition of universities as pivotal in promoting holistic well-being. The lack of consensus and diverse definitions surrounding health and well-being in the academic literature necessitate a systematic approach. The scoping review protocol is designed to develop proposals for measures to improve the health and well-being of university members.

**Objective:**

The objective of this scoping review is to systematically map the domains, topics, and methodological characteristics of survey-based studies assessing health and well-being among university members, including students and employees.

**Methods:**

This protocol follows the PRISMA-ScR (Preferred Reporting Items for Systematic Reviews and Meta-Analyses Extension for Scoping Reviews) guidelines to outline a scoping review to map the existing literature. The review uses the SPIDER (Sample, Phenomenon of Interest, Design, Evaluation, and Research type) tool to define key elements of the research questions. Eligibility criteria include English-language publications reporting on health and well-being surveys of university members published within the past 10 years. Several electronic databases and gray literature repositories were searched.

**Results:**

The scoping review based on this protocol has been completed and published, and this manuscript reports the methodological framework applied during that process. It specifies the primary outputs, including a thematic domain framework, an inventory of survey instruments, and a methodological overview of survey implementation in higher education settings.

**Conclusions:**

By providing a transparent and reproducible methodological description, this protocol supports a comprehensive understanding of health and well-being survey practices in higher education and informs the development of comparable assessment approaches for universities at national and international levels.

**Trial Registration:**

OSF Registries 10.17605/OSF.IO/JMU78; https://osf.io/jmu78/overview

## Introduction

Recently, it has been increasingly recognized and accepted that, alongside health-supporting public policies and care systems, educating individuals about health-conscious lifestyles is essential [1]. Consistent with this, Pronk et al [2] emphasized that the health and well-being of all people is a shared responsibility. According to Dooris and Doherty [3], universities occupy a prominent, privileged, and unique position that can help foster the health and well-being of their members and ultimately society through their policies and practices. Given their multifaceted roles as educational institutions, workplaces, and community actors, universities are increasingly recognized as strategic settings for promoting health and well-being in a comprehensive and sustainable manner [4]. This recognition has been further reinforced by international frameworks and reviews that conceptualize universities as whole systems capable of influencing health through governance, organizational culture, campus environments, and academic activities [5,6].

Universities committed to health promotion integrate health into their daily operations, business practices, and academic pursuits. This integration enhances a university culture steeped in empathy, well-being, equity, and social justice. International literature on health-promoting universities emphasizes that a “whole university” or systems-based approach moves beyond isolated interventions and instead embeds health considerations into institutional strategy, leadership, and decision-making processes [4,7]. Such initiatives not only improve the health of those within the university but also outside the university and strengthen the social, environmental, economic, organizational, and cultural sustainability of both the university community and the surrounding areas [1]. The operationalization of health and well-being development systems has become a crucial aspect of successful university management, profoundly impacting the future trajectory of these institutions [8]. Managing systems to promote health and well-being is now an essential part of running a successful university and is fundamental to the future of universities [6]. This approach necessitates the gathering and analysis of data concerning the health and well-being of the university community, as well as the factors influencing these areas [9,10]. The lack of comprehensive knowledge regarding the health and well-being of university members (ie, students and employees) [11], coupled with the diversity in definitions of the concepts and the ongoing debate in the literature regarding the proper conceptualization and measurement of health and well-being [12], highlights significant challenges in this area. These challenges underscore the need for robust, context-sensitive frameworks and measurement approaches that can inform evidence-based decision-making within higher education institutions (HEIs) [13,14].

According to the Organisation for Economic Co-operation and Development (OECD), the health of the population in Hungary is below what would be expected given its socioeconomic status, and there is a significant gap in opportunities and capabilities for “healthier choices” in daily life [15]. Lifestyle-related risk factors are responsible for almost 50% of deaths in Hungary. Excessive alcohol consumption is high among both adolescents and adults, as are the proportion of obese adults and the rate of preventable deaths. These indicators are worse than the European Union average [15]. Several research studies have been conducted in Hungary examining university students’ health and risk behaviors [16,17] or the methods used to study these behaviors [18,19]. A national survey conducted among Hungarian university students in 2021 (N=7639, 47 universities) examined students’ psychological problems, coping strategies, and resources, drawing attention to the unfavorable psychological state of students (10.2% mental illness, more than 40% symptoms of depression, and more than 50% in crisis) [20]. In 2022, a representative Hungarian research (N=1385) was conducted to examine the risk behavior of university students and compare it with that of a nonuniversity population of the same age. Smoking is less common, while alcohol and drug use are much more prevalent among university students than among nonuniversity students in the same age group. Approximately one-quarter of university students have some chronic physical and/or mental illness and also experience problematic internet use [21,22].

Despite all this, health awareness is not treated as a key issue in Hungary, either in people’s daily lives or at a systemic level. To change this situation, in mid-2023, several academics representing Hungarian universities initiated the establishment of the Network of Health Promotion Universities in Hungary, following the Okanagan Charter [6]. As a first step, they established the Health and Well-being Survey Working Group to develop and implement a joint survey system in line with the World Health Organization (WHO) guidelines to assess the needs and requirements of university members [23]. The authors of this paper, as members of the working group, planned a scoping review to systematically map previous research in this field and to establish a national survey system to support the development of an integrated health and well-being assessment instrument for universities in the network. Scoping reviews are particularly well suited for this purpose, as they allow for the comprehensive mapping of heterogeneous evidence, including variations in conceptual frameworks, target populations, and methodological approaches [24,25]. The review is undertaken to identify the areas covered by past studies, examine their methodological elements, and evaluate the adequacy of their conditions, contributing to a more holistic understanding of health promotion in higher education. By doing so, the authors intend to provide universities with a solid foundation of knowledge and best practices, enabling them to tailor their health promotion strategies effectively. This endeavor is crucial for ensuring that health promotion in universities is evidence-based, contextually relevant, and capable of making a meaningful impact on the health and well-being of university communities. This approach is consistent with the broader literature on health promotion in higher education [3,12], which underscores the need for systematic and comprehensive health initiatives in academic institutions. Furthermore, this review is aligned with the WHO Geneva Charter for Well-being [26] and the global framework for health-promoting schools [23] and adapts these principles to the university context. It also aims to facilitate knowledge transfer across different educational levels, in accordance with WHO guidelines [27]. Despite the growing number of surveys, no scoping review has systematically mapped the domains, instruments, and methodological characteristics of university-based health and well-being surveys. A scoping review conducted using the methodological framework described in this protocol has since been completed and published [28].

On the basis of our study objectives, we propose a protocol that is specifically tailored for universities embarking on initiatives to promote health. This scoping review, serving as the initial phase of the planned survey system, aims to examine health-related and well-being–related surveys performed in universities. The goal is to explore which measurement tools are used and which aspects of well-being are assessed.

## Methods

### Reporting Guidelines

This protocol is reported in accordance with the PRISMA-P (Preferred Reporting Items for Systematic Reviews and Meta-Analyses Protocols) 2015 guidelines. The completed scoping review derived from this protocol followed the PRISMA-ScR (Preferred Reporting Items for Systematic Reviews and Meta-Analyses Extension for Scoping Reviews) reporting guidelines [29]. The methodological approach is described below. This protocol documents the methodological framework applied in the completed scoping review [28] and is presented to support transparent reporting and reuse in future scoping reviews in this field.

The protocol was registered in the Open Science Framework (OSF) Registries.

### Rationale

As mentioned in the Introduction, our Surveying Health and Well-being Working Group is designing a survey system for assessing the health and well-being of university members, which is intended to be developed based on the information currently available in the literature. Ideally, the information obtained from the survey system may inform the planning and implementation of interventions by Hungarian universities to improve the health and well-being of their members. It is envisaged that the survey system will consist of a questionnaire, a protocol for the implementation of the survey, consultative advice, a common database, analysis procedures, and reporting templates. Universities wishing to use the survey system will agree to adhere to the survey protocol in an agreement with the working group, which is intended to support comparability of results between universities and analysis of national or university trends. For the development of the survey system, this scoping review is intended to collect topics (possibly questions) that have already been used to assess health and well-being, and methodological experience in the design, preparation, implementation, and evaluation of relevant surveys on this topic.

In addition to its applied relevance, this scoping review is intended to support a conceptual synthesis of how health and well-being are operationalized and measured in higher education contexts. By mapping the domains, constructs, and indicators assessed across existing surveys, the review will contribute to the identification of conceptual patterns and gaps in the current measurement landscape. This domain-based mapping allows the findings to be interpreted in relation to international frameworks of health and well-being used in public health and educational research. In this way, the review will provide a conceptual foundation that complements its potential applied and policy-relevant implications.

### Objectives

Our scoping review was designed to answer the following two questions: (1) What topics are covered in the articles on university members’ health and well-being? and (2) What methodologies and techniques are used in the health and well-being surveys of the university members?

The purpose of this scoping review is to systematically map the domains, instruments, and methodological characteristics of health and well-being surveys conducted in higher education. This mapping provides a structured basis for understanding how health and well-being are currently operationalized and measured in university settings.

### Eligibility Criteria

As Table 1 describes, the eligibility criteria were developed in accordance with the general practice of systematic reviews and with the time and human resources available to us. All types of English-language publications published in the past 10 years that meet the search criteria and are available in full text were considered. In this way, the researchers were able to gather as much information as possible on the topics to be considered in the development of the questionnaire and the design of the survey protocol.

According to the literature [30], the SPIDER (Sample, Phenomenon of Interest, Design, Evaluation, Research Type) tool is appropriate for scoping reviews. This was used to define key elements of the review research question. The sample (the target population) was university members in line with the aims of our project. The phenomena studied were all types of research that aim to measure the health and well-being of university members. Design and evaluation were not restricted; that is, anything could be used, as the topics to be studied could be drawn from any research. For the research type, the review focused on surveys.

**Table 1:** Eligibility criteria for the review. The criteria were organized following the SPIDER (Sample, Phenomenon of Interest, Design, Evaluation, Research Type) approach. Eligibility was further informed by the World Health Organization’s multidisciplinary framework on well-being [23].

Category	Inclusion criteria	Exclusion criteria
Sample	Adults (aged 18 years and above), university members (students and staff), including mixed populations when general health and well-being were assessed^a^	Non-English studies
Phenomenon of Interest	All types of research that aim to measure the health and well-being (physical, mental, and social) of university members	Studies conducted more than 10 years ago
Design and Evaluation	Not restricted	No full text available in the public domain
Research type	Quantitative questionnaire study	Studies focusing exclusively on disease prevalence or unrelated academic outcomes without an explicit connection to well-being

^a^Studies including university members with and without self-reported health conditions were eligible if the primary focus was the assessment of general health and/or well-being domains at the population level rather than disease-specific outcomes.

### Information Sources

The following electronic databases were searched for both published and unpublished (gray) literature on the research topic, covering the period from January 1, 2014, to May 31, 2024: PubMed, Cochrane Library, Scopus, Web of Science, and Google Scholar. These databases were chosen as they are suitable for conducting Boolean searches and for the transparent ranking of results. For gray literature, the search included OSF, PrePrints, SSRN, and Preprints with The Lancet, as well as institutional and organizational repositories containing reports and survey data on health and well-being in higher education (eg, international and national public health and higher education agencies).

Gray literature items were assessed using the AACODS (Authority, Accuracy, Coverage, Objectivity, Date, and Significance) checklist to ensure the relevance and quality of nonpeer-reviewed sources.

### Search Strategy

The search strategy was structured in accordance with the Population, Concept, and Context (PCC) framework recommended for scoping reviews. The population comprised university members (including students and employees), the concept focused on health and well-being as assessed through surveys, and the context was HEI. On the basis of this framework, search terms were developed and combined using Boolean operators. Derived from our research questions, the initial concepts and the related term labels for constructing the actual search phrase are as follows:

- HEI-related term labels are *university* and *college*
- Term label for members of HEIs, including students (related term labels: *undergraduate*, *graduate and postgraduate student*, *bachelor, master, doctorandus*, and *PhD student*) and employees (related term labels: *staff, personnel, workers*, and *teachers*).
- Health (related term labels: *wellness* and *fitness*)
- Well-being (related term labels: *happiness*, *welfare*, and *quality of life*)
- Survey (related term labels: *assessment, investigation, analysis, exploration*, and *questionnaire*)

The Boolean logical buildup of concepts for searching is as follows: *HEI* AND *member* AND (*health* OR *well-being*) AND *survey*

To build a comprehensive and focused search phrase, we took the following steps. First, to find all relevant synonyms and conceptually related terms, we used the Unified Medical Language System, the largest available health-related metathesaurus, containing well over 4.5 million lexical entities. Second, we developed the search strategy using the PCC framework to systematically define and combine key search terms. Third, on the basis of the result of the step “selection of bibliographic sources,” we translated the Boolean expression to the syntax required by the bibliographic source. Fourth, we conducted pilot searches in the selected databases and examined the keywords and index terms (if applicable) of the top 30 most relevant papers. This step was an iteration to refine the search phrase and increase its precision; the results are already in the list of labels above. Fifth, we expanded the search phrase as needed, translated it to the source syntax again, and performed the final searches.

It must be noted that concepts are mentioned above by their labels (descriptive words) themselves—used in the search phrasing as well. The first search phrase we tried is shown in Textbox 1.

Initial overview showed that such a search was not specific for intrainstitutional surveys, resulting in tens of thousands of (mostly nonrelevant) papers. After several pilot searches, the search phrase was finalized to the one in Textbox 2, which was used in all the databases selected.

This broad scope of interest, even with this refined search, resulted in more than several thousand studies to screen. Due to time and resource constraints frequently encountered in practice, the screening process was limited to a subset of the highest-ranked records in each database. Accordingly, this scoping review protocol was conducted as a rapid scoping review. Rather than performing an exhaustive screening of all retrieved records, a structured and transparent sampling approach was applied. In each database, the screening was limited to the highest-ranked records returned by the database-specific relevance algorithms. This approach was chosen to balance feasibility with the objective of identifying the most relevant survey-based studies and is consistent with pragmatic adaptations described in the methodological literature on rapid evidence syntheses. The limitations associated with this approach, including potential algorithmic bias and reduced reproducibility, were acknowledged and considered in the interpretation of the findings.

**Initial search phrase.**

- Population: (“member*” OR “student*” OR “employ*” OR “undergraduat*” OR “graduat*” OR “postgraduat*” OR “bachelor*” OR “master*” OR “doctorand*” OR “staff” OR “personnel” OR “workers” OR “teachers” OR “lecturer*”)
- Concept: AND (“health” OR “wellness” OR “fitness”) AND (“well-being” OR “happiness” OR “welfare” OR “quality of life”)
- Context: AND (“higher education institute*” OR “universit*” OR “colleg*”)
- Extra (outcome): AND (“survey” OR “assess*” OR “investigat*” OR “analys*” OR “explor*”)

**Final search phrase.**

- Population: (student OR staff OR employee OR member OR personnel OR worker OR administrator)
- Concept: AND (“health” OR well-being OR wellbeing)
- Context: (“in university” OR “at university” OR “in college” OR “at college”)
- Extra (outcome): AND (survey or questionnaire)

### Selection of Sources of Evidence (Screening)

To select the sources, the following steps were performed. First, a deduplication process identified and removed duplicate hits from the search results using EndNote 20 (Clarivate). The screening phase then began with an initial assessment based on the titles and keywords of the identified articles. This was followed by a detailed evaluation of the abstracts. Each article was independently screened by 2 reviewers who excluded studies that were clearly outside the predefined scope of the review. Any discrepancies or disagreements were resolved by consensus and in consultation with a senior researcher. The screening phase was completed within 6 weeks.

### Data Charting Process (Extraction)

Citations from each search were downloaded into reference manager software (EndNote), where duplicates were identified and removed. To ensure the degree of agreement among independent observers is appropriate, interrater reliability was calculated using Cohen κ, with a threshold of 0.8, using freely available online or other statistical software (Covidence). Interrater agreement was also assessed and reported using percentage agreement, depending on the screening tool applied by the implementing research team. Also, the 0.8 threshold was intended as a recommended target to guide interrater reliability rather than as a rigid exclusion criterion. The reviewers then evaluated the eligible studies and extracted the information in accordance with predefined criteria listed in the Data Items section. Eligible studies were entered into a study database using a standardized data extraction template developed in Microsoft Excel. The data extraction form was pilot-tested on a predefined subset of studies to calibrate the interpretation of data items, and the form was iteratively refined based on insights gained during this pilot phase. To ensure accuracy, the information extracted by each reviewer was compared and discrepancies were discussed to ensure consistency. Disagreements were resolved through discussion and, when necessary, consultation with an independent third-party senior researcher.

### Data Items

Each publication was assessed according to the following criteria.

The following topics were covered by the survey:

- Health status characteristics (physical and/or mental health), well-being characteristics, and health behaviors.
- Influencing factors, including individual or family socioeconomic status, living environment, working or studying environment, social networking, work–study life balance, and other relevant factors.

The following methodological characteristics were used for these surveys:

- Data collection methods, including online, face-to-face, and phone-based approaches
- Data collection tool, including paper-pencil or computer-based instruments
- Purpose of data collection, including screening or health assessment
- Target population, including students and/or employees
- Sampling procedure and sample size
- Survey questions, including the use of validated instruments
- Evaluation methods

Textbox 3 presents how the SPIDER framework was operationalized in this review by mapping each SPIDER element to the corresponding data items extracted.

**Operationalization of the SPIDER (Sample, Phenomenon of Interest, Design, Evaluation, Research Type) framework in data extraction.**

**Sample**

- University members (students and/or employees), as defined in the eligibility criteria

**Phenomenon** **of interest**

- Health and well-being topics assessed in surveys, including health status, well-being characteristics, health behaviors, and influencing factors

**Design**

- Survey-based study designs

**Evaluation**

- Measurement domains and survey instruments extracted under Data Items

**Research type**

- Survey-based research

### Synthesis of Results

As an initial step, the information extracted from the evaluation of the publications was synthesized in a narrative manner and, where relevant, through descriptive statistics. The findings were summarized according to the topics surveyed and the methodology used. The result of this synthesis process was a thematic domain framework of health and well-being constructs assessed across surveys; an inventory of survey instruments and items; and a methodological and implementation feature map describing survey designs, sampling approaches, and modes of administration used in higher education settings.

## Results

**Figure 1:**
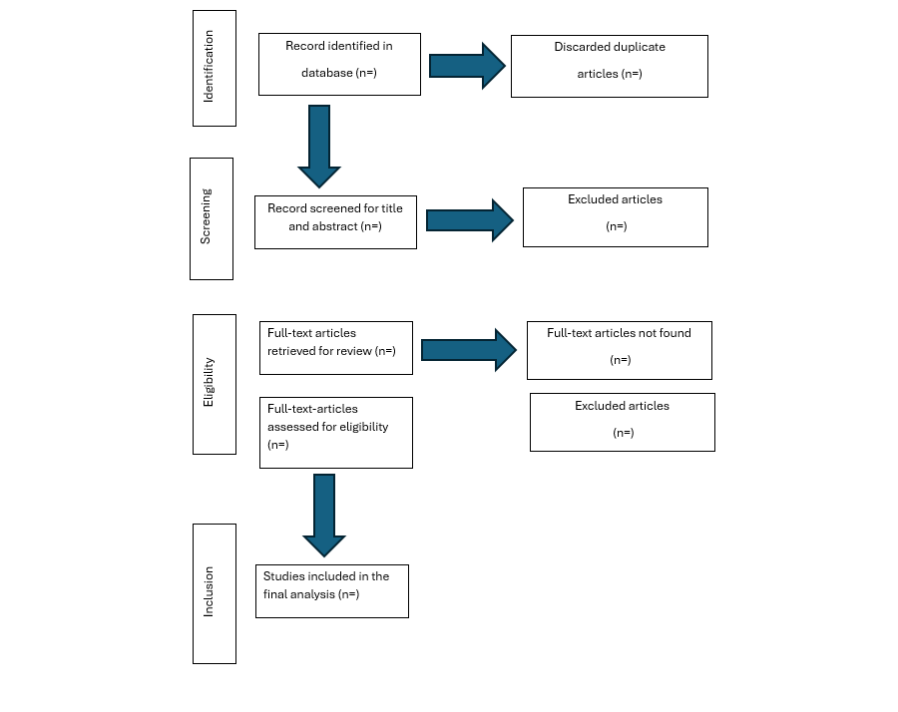
PRISMA (Preferred Reporting Items for Systematic Reviews and Meta-Analyses) flow diagram summarizing the identification, screening, and inclusion of studies in the completed scoping review.

The first scoping review to which this protocol was applied has been completed, and the results have been published [28]. Drawing on the empirical and methodological insights of that review, this protocol outlines a structured approach for future scoping reviews in this field. It proposes the development of a thematic domain framework, an inventory of survey instruments, and a methodological overview of survey practices in higher education. A PRISMA (Preferred Reporting Items for Systematic Reviews and Meta-Analyses) flow diagram will be used to summarize the identification, screening, and inclusion of studies (Figure 1).

## Discussion

### Anticipated Findings

This scoping review aimed to bridge gaps in knowledge regarding how health and well-being are assessed through surveys conducted among university members. The protocol follows scoping review methodology and relevant guidelines, ensuring a systematic and comprehensive examination of health and well-being surveys in universities. By clearly defining the scope, eligibility criteria, and data extraction strategy, the protocol supports methodological consistency and reproducibility in the synthesis of health and well-being surveys conducted in universities. As the research addresses a timely and crucial issue, emphasizing the role of universities in promoting health and well-being, the protocol may inform practical applications in the field of university health and well-being assessment. The published scoping review reports the empirical findings of applying this framework [28], while this protocol provides a detailed methodological description intended to support replication and future applications. It is important to note that minor methodological refinements were introduced between the protocol and the published review (eg, SPIDER-based structuring of eligibility criteria, PCC-based structuring of the search strategy, specification of the time frame, explicit framing as a rapid scoping review, inclusion of AACODS criteria for gray literature appraisal, and more detailed procedures for data extraction). These refinements do not alter the underlying methodological approach described in the protocol.

### Limitations

A limitation of the protocol is that it restricts its inclusion criteria to publications after 2014 (the past 10 years). Although the 10-year time frame was retained as it reflects a commonly used research practice for defining up-to-date literature in rapidly developing fields, it potentially omits relevant earlier research. Furthermore, focusing solely on English-language publications may introduce a language bias, excluding valuable insights from non-English sources. Restricting inclusion to English-language publications was driven by feasibility and resource constraints, including time limitations and the lack of multilingual screening capacity within the review team. This restriction may result in an overrepresentation of English-speaking and Western European perspectives and an underrepresentation of approaches and survey practices relevant to the Hungarian and Central and Eastern European context. Although artificial intelligence–assisted translation tools could theoretically broaden the scope of included studies, their use entails a risk of translation inaccuracies that require expert linguistic review. In the absence of sufficient language proficiency and human resources to reliably identify and correct such errors, this approach was not adopted. The decision to limit screening to a subset of records due to resource constraints represents a methodological limitation that may affect the comprehensiveness of the review. These limitations should be considered when interpreting the methodological scope and transferability of the findings. As this review is exploratory in nature and aims to map domains, instruments, and methodological characteristics of survey-based research, the strength of the body of evidence was not formally assessed using tools such as Grading of Recommendations Assessment, Development and Evaluation (GRADE).

### Conclusions

This scoping review protocol outlines a systematic approach to mapping health and well-being surveys conducted among university members. By defining clear eligibility criteria, search strategies, and data extraction procedures, the protocol aims to support a transparent and reproducible synthesis of existing survey-based evidence. The findings derived from this protocol are expected to contribute to a clearer understanding of how health and well-being are operationalized and measured in higher education contexts, and to inform future methodological and conceptual developments in this field.

## Abbreviations

AACODS: Authority, Accuracy, Coverage, Objectivity, Date, and Significance
GRADE: Grading of Recommendations Assessment, Development and Evaluation
HEI: higher education institution
OECD: Organisation for Economic Co-operation and Development
OSF: Open Science Framework
PCC: Population, Concept, and Context
PRISMA: Preferred Reporting Items for Systematic Reviews and Meta-Analyses
PRISMA-P: Preferred Reporting Items for Systematic Reviews and Meta-Analyses Protocols
PRISMA-ScR: Preferred Reporting Items for Systematic Reviews and Meta-Analyses Extension for Scoping Reviews
SPIDER: Sample, Phenomenon of Interest, Design, Evaluation, Research Type
WHO: World Health Organization

## Data Availability

All data generated or analyzed during this study were included in the published scoping review article.
